# Protein Binding Detection Using On-Chip Silicon Gratings

**DOI:** 10.3390/s111211295

**Published:** 2011-11-28

**Authors:** Anil Kumar Mudraboyina, Jayshri Sabarinathan

**Affiliations:** Department of Electrical and Computer Engineering, the University of Western Ontario, London, ON N6A 5B9, Canada; E-Mail: amudrabo@uwo.ca

**Keywords:** gratings, immobilization, silicon, biosensor, protein

## Abstract

We demonstrate a silicon gratings-based biosensor to detect functionalized protein binding on its surface. The designed silicon gratings have sensitivities up to 197 nm/RIU in detecting refractive index change and 1.61 nm per nanometer of thickness change of bio-material on the surface of silicon gratings. Functionalizing proteins on gratings surface by eliminating unspecific binding makes this device more selective and efficient. Streptavidin at a concentration of 0.016 μmol/mL was functionalized on silicon substrate and biotin of 12 μmol/mL concentration was used as a target molecule in our detection experiments. Normal transmission measurements of gratings are made in air at different stages of immobilization, bare silicon grating, after attaching streptavidin and after trapping biotin. Total shifts in resonant peak wavelength of ∼15 nm in normal transmission were observed after immobilizing biotin with ∼7 nm of shift in resonant peak wavelength after functionalizing streptavidin to silicon substrate.

## 1. Introduction

A grating is an optical component with a periodic dielectric pattern in one dimension, which when it consists of sub-wavelength features and has thin-film layers, shows resonance spectral characteristic [1]. More generally a grating is a dispersive element commonly used in monochromators and spectrometers. Apart from these applications, on-chip gratings are also used for spectral shaping [2], mode converters in optical communications [3], narrow band optical filters [4] and chemical/biological sensors [5–14]. Resonant gratings, being highly sensitive to the refractive index of the material in its surroundings, have attracted many researchers to explore their sensing applications. These sensors are typically coated with materials of nano-scale thicknesses to observe the spectral changes [6–14]. Gratings are compatible with building fluid chambers for biosensing purpose, where the device response to the bio-material with respect to time measurement can be investigated [10].

In this paper, we demonstrate gratings on silicon for streptavidin-biotin detection by eliminating unspecific bindings which makes this device more selective and efficient. These gratings are built on-chip, which makes it more compact, results in faster response and is easy for immobilization compared to gratings on optical fibers [5–9,11–13]. It has been shown that the sensitivity of fiber grating sensors is around three times higher when measurements are made in air rather than in liquids [12] using a fluid chamber over gratings to detect analyte in fluid media [10]. Specific binding on the sensor surface is a key feature in sensor applications since this avoids spectral shifts due to unspecific binding as in the case of simple physical absorption or coating of analyte on the surface of gratings [5–8]. Here, we demonstrate a sensor using silicon gratings with streptavidin immobilized on its surface through ionic bonding which has greater affinity towards biotin. By detecting only specific binding, the sensor measurements can be done in air rather than in fluid chambers for sensitive detection. Shifts in resonant peak wavelengths in normal transmission were estimated through simulations using a 3-D finite-difference time domain (FDTD) method with non-uniform mesh. Gratings were fabricated by standard e-beam lithography. Wei Zhang *et al*. have demonstrated polyester gratings with TiO_2_ nanorods incorporated to increase sensitivity, but fabrication of these structures is complicated and they have issues with repeatability [14]. Here, we have selected the design in such a way that these silicon gratings are simple to fabricate and repeatable. The small footprint of the silicon grating and small quantity and concentration of analyte required, make these silicon gratings an attractive sensor with high sensitivity. Normal transmission of these devices were recorded at different stages—bare silicon gratings, after functionalizing the surface with streptavidin and after biotin binding—to observe shifts in resonant peak wavelength. Characterization on this bio-material layer was done for its uniformity, thickness and for the uniform distribution of proteins on grating surface using AFM imaging, ellipsometer and confocal imaging to locate proteins.

## 2. Simulations

**Figure 1.**
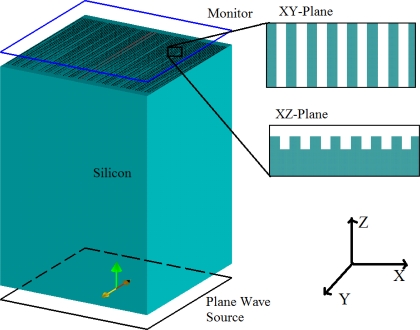
Gratings structure model used in the Lumerical FDTD simulation software.

Lumerical’s FDTD solution software was used to numerically simulate our grating structure. The gratings were constructed on a 425 μm thick silicon slab with periodicity of 630 nm, groove width and height of 300 and 400 nm, respectively. In order to avoid huge computations due to the dimensions of these devices, the FDTD method was used with periodic boundary conditions in the X, Y directions. We implemented a full 3-D FDTD simulation based on square non-uniform mesh since a major part of this device is a silicon substrate with shallow gratings on its surface. Based on this technique, the default grid size used to mesh the structure was set at 18 nm with a finer 10 nm mesh over the gratings pattern region. In this way, the accuracy and convergence of the simulations are both guaranteed, while the computational resource requirements are reasonable enough to run the simulation. The light source was placed at the bottom of the silicon slab and normal transmission was recorded at the top of the gratings. Figure 1 shows the simulation model built with the Lumerical FDTD simulation software.

The resonant peak was observed at 1,135 nm. There are two reasons for designing the resonant peak at this wavelength. First, silicon has a strong absorption in the visible and near infrared region up to 1,000 nm, so any grating designs on silicon for transmission measurements must have a transmission resonance peak greater than 1,000 nm. Secondly the InGaAs detector used in the measurements has a maximum spectral response this region.

**Figure 2.**
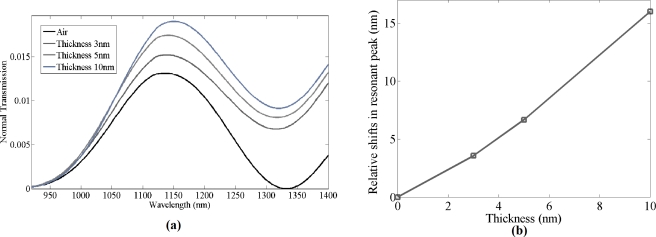
Simulation results: **(a)** Shifts in resonant peak due to bio-material coatings of different thickness on silicon gratings; **(b)** Shifts in resonant peak wavelengths with respect to thickness of bio-material.

Once the bare grating structure was successfully simulated, a thin film of material with refractive index 1.502 was simulated on top of the gratings with different thicknesses ranging from 3 to 10 nm. The material was set to have same optical properties as the biochemicals used in the experiment. Grating structures with different bio-material thickness were simulated to estimate the shifts in resonant peak wavelength. Normal transmission spectra for thickness of 3–10 nm of bio-material on top of the gratings were simulated. As seen in Figure 2(b), the resonant peak wavelength red shifts as the thickness of the bio-material increases. When the estimated thickness of the bio-material layer was ≈10 nm, the resonant peak wavelength shift in the simulated data was observed to be 16 nm, with a sensitivity of 1.60 nm per nanometer of thickness change of bio-material of refractive index 1.502 on the grating surface, which is higher compared to photonic crystal micro-cavity bio-sensors [15]. Zhiyong Wang *et al.* have reported similar sensitivity for optical fiber gratings, but for detecting a higher refractive index material [9].

## 3. Fabrication

**Figure 3.**
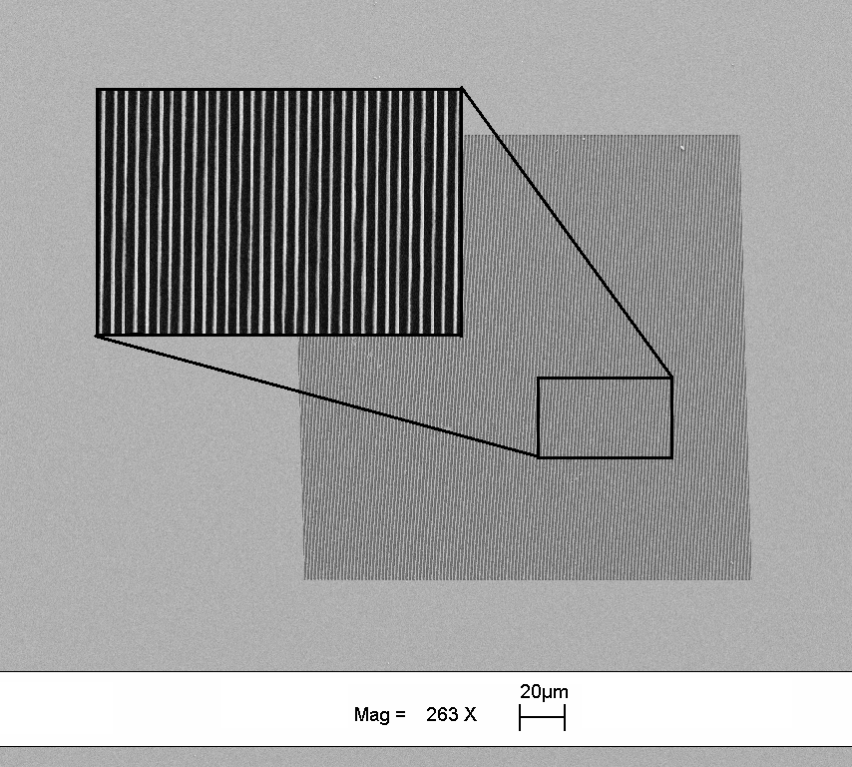
SEM image of silicon gratings fabricated using e-beam lithography.

The gratings were fabricated on a double side polished silicon wafer of 425 μm thickness. The grating pattern was written on ZEP-520a resist on a silicon substrate using e-beam lithography. The samples were developed in ZED-N50 and the silicon was etched using a deep silicon plasma etcher with an etch rate of 12.5 nm/s for 32 s. After successful etching, the samples were cleaned in an oxygen plasma asher to remove hydrocarbon contaminants and any resist remaining during fabrication and gratings depth was measured to be ∼400 nm. The dimensions of the fabricated gratings were chosen to be 200 × 200 μm (length × width) and the period of the grating was fixed at 630 nm. Figure 3 shows the SEM image of the gratings fabricated on double sided polished silicon wafer.

Gratings with different groove width were fabricated by varying the e-beam dose. The groove widths of silicon gratings were later measured accurately using an AFM and found to vary from 359 to 480 nm. Normal transmission through the gratings with different groove widths are expected to have resonant peaks at different wavelengths as the gratings resonant peak is highly sensitive to the grating width, grating depth and periodicity [16,17].

## 4. Immobilization and Characterization

### 4.1. Immobilization of Streptavidin and Biotin on Grating Surface

**Figure 4.**
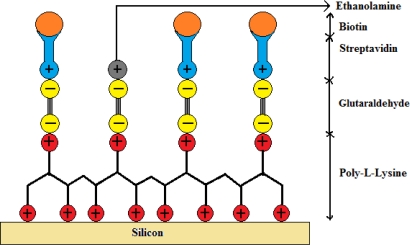
Schematic of the immobilization process on silicon substrate.

The immobilization technique used in this work was adapted from a previously published process on photonic crystal fibers for labeled antibody detection [18]. In our experiment the immobilization technique was modified with biotin as a target molecule instead of antibodies to make it a label free sensor. Prior to immobilizing the streptavidin on gratings, a biochemical coating of poly-l-lysine and glutaraldehyde is deposited to ensure trapping of the streptavidin on the gratings. Between each step of the coating procedure, the gratings were rinsed in a pH neutral solution of Phosphate Buffered Saline (PBS, 10 mmol) for 5 min. The first layer was deposited on silicon by immersing the samples in an aqueous solution of poly-l-lysine (1:100 in H_2_O) acting as a positively charged substrate for 15 min, covered then by a negatively charged layer of glutaraldehyde (12.5%) for 45 min which then binds to the poly-l-lysine. The samples were then left in streptavidin (0.016 μmol/mL in PBS of neutral pH) for 30 min to bind to the glutaraldehyde sites. Finally, the empty sites of glutaraldehyde were blocked by positively charged ethanolamine molecules (40 mmol) by keeping the samples immersed for 20 min. A schematic of the biochemical layers holding the streptavidin and biotin is shown in Figure 4. A subsequent PBS washing eliminated the unspecific bindings and addition of ethanolamine molecules covered up the unbounded sites of glutaraldehyde preventing any unspecific binding. Streptavidin is a protein with an exceptionally high binding constant of 10^15^ M^−1^ with biotin. The biotin-streptavidin system serves as the bio-conjugate pair in this demonstration. Once the streptavidin was functionalized on to the silicon substrate, the samples were covered with biotin (12.27 μmol/mL in PBS of neutral pH) for 30 min and samples were rinsed with PBS solution to remove any unbound biotin from the silicon surface.

### 4.2. Characterization of Biochemical Layer with Streptavidin and Biotin on Gratings Surface

**Figure 5.**
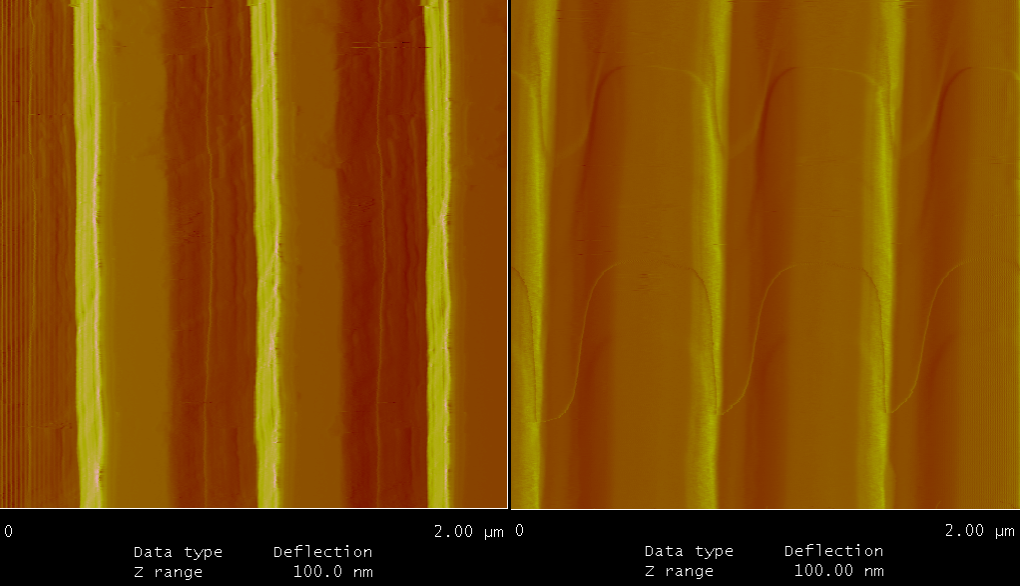
AFM images of grating surface before and after immobilization.

The uniformity, thickness of bio-material on the grating surface were characterized using AFM imaging and ellipsometry. Figure 5 shows the AFM images of the bare silicon gratings and after biotin immobilization on the grating surface. The samples are uniformly covered with the bio-material and uniformity of this layer was measured to be ±0.48 nm on the total bio-material layer thickness of 9.25 nm. The left image in Figure 5 shows the sharp edges of the silicon grating whereas the image on the right shows the biochemical layer with streptavidin and biotin uniformly covering the inner walls and the top surface of the gratings. Ellipsometry was used to measure the thickness of the biochemical layer along with streptavidin and biotin. In addition to thickness measurements, ellipsometry was also used to measure the refractive index of the layers coated on silicon surface. The thickness of the biochemical layer along with streptavidin and biotin was measured to be 9.25 ± 0.25 nm and the refractive index was found to be 1.502 at 1,137 nm. Ellipsometry measurements were done on bare silicon as a grating pattern can interact with the polarized light used for thickness measurements and refractive index measurement. AFM shows conformal coverage and hence ellipsometry on bare silicon should yield accurate results.

## 5. Experimental Measurement Results and Discussion

**Figure 6.**
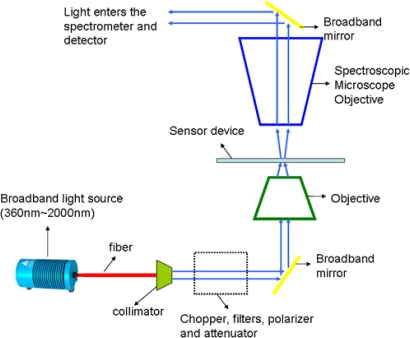
Schematic of measurement setup.

Normal transmission measurements were performed on the gratings using 0.8 m high resolution spectrograph and InGaAs detector. As shown in Figure 6, the measurement setup consists of broadband light source from 360–2,000 nm which was passed through a collimator. The collimated beam was then passed through a series of optical components such as chopper, filters, polarizer and attenuator. Polarized light was then focused using an objective onto the sample. Transmitted light from the sample was collected by a spectroscopic microscope objective and then directed into the spectrometer and detector.

**Figure 7.**
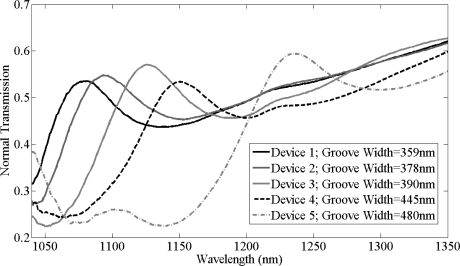
Normal transmission spectra of grating devices with different groove widths.

Initially, normal transmission measurements were recorded from five bare silicon gratings with different groove widths. Transmission spectra of these bare silicon gratings, as shown in Figure 7, have different resonant peaks due to the different fabricated groove widths of these gratings. AFM was used to measure the groove width of these gratings which were as labeled in Figure 7. The resonant peak wavelength red shifts with increase in groove width of these gratings. Device 1, device 2, and device 5 [dev(1,2,5)] with groove widths of 359, 378 and 480 nm respectively were used to study the specific binding of biotin on the silicon grating surface while device 3 and device 4 [dev(3,4)] with groove widths of 390 and 445 nm were used to observe the effects of unspecific binding.

**Figure 8.**
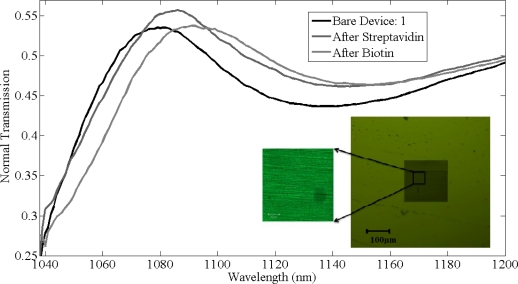
Measurements recorded on device 1 at different stages of immobilization with inset of confocal image of the device after functionalizing with biotin where the streptavidin was tagged with fluorescein.

The thickness of chemical layer of after coating poly-l-lysine and glutaraldehyde was found to be approximately half a nanometer with refractive index 1.35 and the transmission shift produced in normal peak of gratings is negligible. The thickness of the bio-material layer after immobilizing streptavidin on the silicon gratings is expected to be around 5 nm. After functionalizing streptavidin on the gratings surface, measurements were recorded and a shift of 7 ± 1 nm was observed in the resonant peak in all the gratings. Final measurements were recorded after biotin binding to streptavidin. Dev(1,2,5) were washed with PBS solution to remove the unbounded biotin where as dev(3,4) are unwashed samples. Figure 8 shows the shifts in the resonant peaks of the normal transmission spectra due to streptavidin and biotin binding in device 1, with the inset showing the confocal image of the device after functionalization with biotin. Streptavidin was tagged with fluorescein with fluorescence signal at 514 nm confirms the presence of streptavidin binding on silicon grating surface.

**Figure 9.**
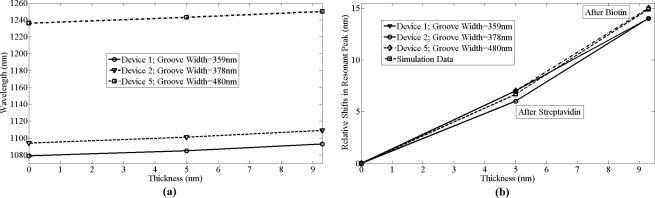
**(a)** Shifts in resonant peak due to immobilization on different gratings sensors with respect to thickness of bio-material; **(b)** Comparison of simulation data with measurement results.

For dev(1,2,5), shifts of 8 ± 1 nm in resonant peak wavelength were observed due to specific binding of biotin to streptavidin, as shown in Figure 9. In comparison, for dev(3,4) shifts of 110 nm and 95 nm in resonant peak wavelength was observed, respectively, due to the physical adsorption of biotin along with biotin that was bonded to streptavidin. There is a difference of 1 nm in shifts among dev(1,2,5) after functionalizing with biotin as the immobilized layer after biotin varied by ±0.25 nm for these devices. Figure 9(a) shows the absolute shifts in wavelength with increasing groove width as well as wavelength response of dev(1,2,5) to streptavidin and biotin binding, while Figure 8(b) shows the relative shifts in resonant peak wavelength due to streptavidin and biotin functionalization on the grating surface. Though the streptavidin is bigger in size compared to biotin, due to the concentration difference between streptavidin and biotin, more binding sites for biotin on streptavidin are available and also one of the important factors is the refractive index of each bio-material layer, which produces the resonant peak shift. It is not possible to measure the refractive index of each and every layer individually. The measured refractive index of 1.502 is the effective refractive index after immobilizing streptavidin and biotin. It was observed from Figure 9(a,b) that the device resonant peak wavelength response to streptavidin and biotin binding remains constant and independent of original groove width differences. The simulation prediction matches closely with the measurement data and the immobilization technique in this experiment was designed so as to functionalize monolayers of streptavidin and biotin to have a better control of the thickness and distribution of streptavidin and biotin on the silicon surface. We used 12.27 μmol/mL of biotin for 0.016 μmol/mL of streptavidin for our experiment and sensitivity of 1.61 nm per a nanometer of thickness change of bio-material on the surface of silicon gratings was measured. This is a large concentration and there is probably still some non-uniformity as they are coated on the gratings and the optical measurement is averaged over a larger area. So while the spectral shifts are confirmed repeatedly, the exact thickness of the added layer has some uncertainty (also due to refractive index changes mentioned earlier). Further measurements with different concentrations have to be investigated.
